# Impact of remission from type 2 diabetes on long-term health outcomes: findings from the Look AHEAD study

**DOI:** 10.1007/s00125-023-06048-6

**Published:** 2024-01-18

**Authors:** Edward W. Gregg, Haiying Chen, Michael P. Bancks, Raoul Manalac, Nisa Maruthur, Medha Munshi, Rena Wing, for the Look AHEAD Research Group

**Affiliations:** 1 School of Population Health, Royal College of Surgeons of Ireland, Dublin, Ireland; 2 Department of Epidemiology and Biostatistics, School of Public Health, Imperial College London, London, UK; 3 Wake Forest University School of Medicine, Winston-Salem, NC USA; 4 Pennington Biomedical Research Center, Baton Rouge, Louisiana USA; 5 Division of General Internal Medicine, Johns Hopkins University School of Medicine, Baltimore, MD USA; 6 Joslin Diabetes Center, Division of Gerontology, Beth Israel Deaconess Medical Center, Harvard Medical School, Boston, MA USA; 7 Department of Psychiatry and Human Behavior, Alpert Medical School of Brown University, Providence, RI USA

**Keywords:** Cardiovascular disease, Chronic kidney disease, Diabetes, Lifestyle intervention, Remission, Weight loss

## Abstract

**Aims/hypothesis:**

We examined the association of attainment of diabetes remission in the context of a 12 year intensive lifestyle intervention with subsequent incidence of chronic kidney disease (CKD) and CVD.

**Methods:**

The Look AHEAD study was a multi-centre RCT comparing the effect of a 12 year intensive lifestyle intervention with that of diabetes support and education on CVD and other long-term health conditions. We compared the incidence of CVD and CKD among 4402 and 4132 participants, respectively, based on achievement and duration of diabetes remission. Participants were 58% female, and had a mean age of 59 years, a duration of diabetes of 6 year and BMI of 35.8 kg/m^2^. We applied an epidemiological definition of remission: taking no diabetes medications and having HbA_1c_ <48 mmol/mol (6.5%) at a single point in time. We defined high-risk or very high-risk CKD based on the Kidney Disease Improving Global Outcomes (KDIGO) criteria, and CVD incidence as any occurrence of non-fatal acute myocardial infarction, stroke, admission for angina or CVD death.

**Results:**

Participants with evidence of any remission during follow-up had a 33% lower rate of CKD (HR 0.67; 95% CI 0.52, 0.87) and a 40% lower rate of the composite CVD measure (HR 0.60; 95% CI 0.47, 0.79) in multivariate analyses adjusting for HbA_1c_, BP, lipid levels, CVD history, diabetes duration and intervention arm, compared with participants without remission. The magnitude of risk reduction was greatest for participants with evidence of longer-term remission.

**Conclusions/interpretation:**

Participants with type 2 diabetes with evidence of remission had a substantially lower incidence of CKD and CVD, respectively, compared with participants who did not achieve remission. This association may be affected by post-baseline improvements in weight, fitness, HbA_1c_ and LDL-cholesterol.

**Trial registration:**

ClinicalTrials.gov NCT00017953

**Data availability:**

https://repository.niddk.nih.gov/studies/look-ahead/

**Graphical Abstract:**

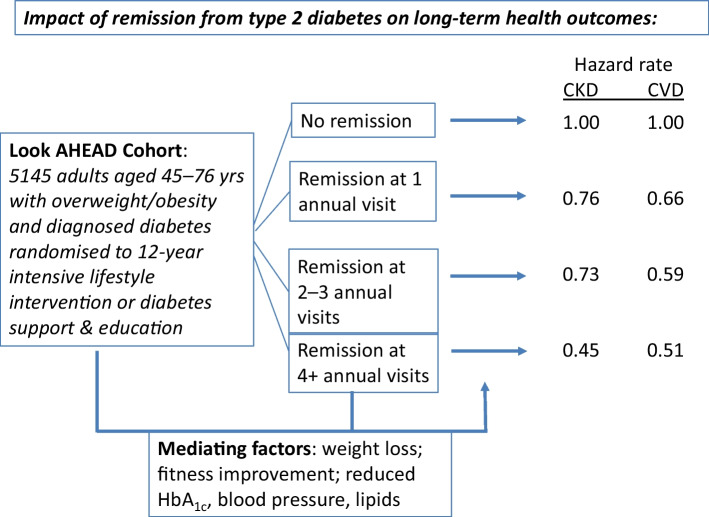

**Supplementary Information:**

The online version of this article (10.1007/s00125-023-06048-6) contains peer-reviewed but unedited supplementary material.


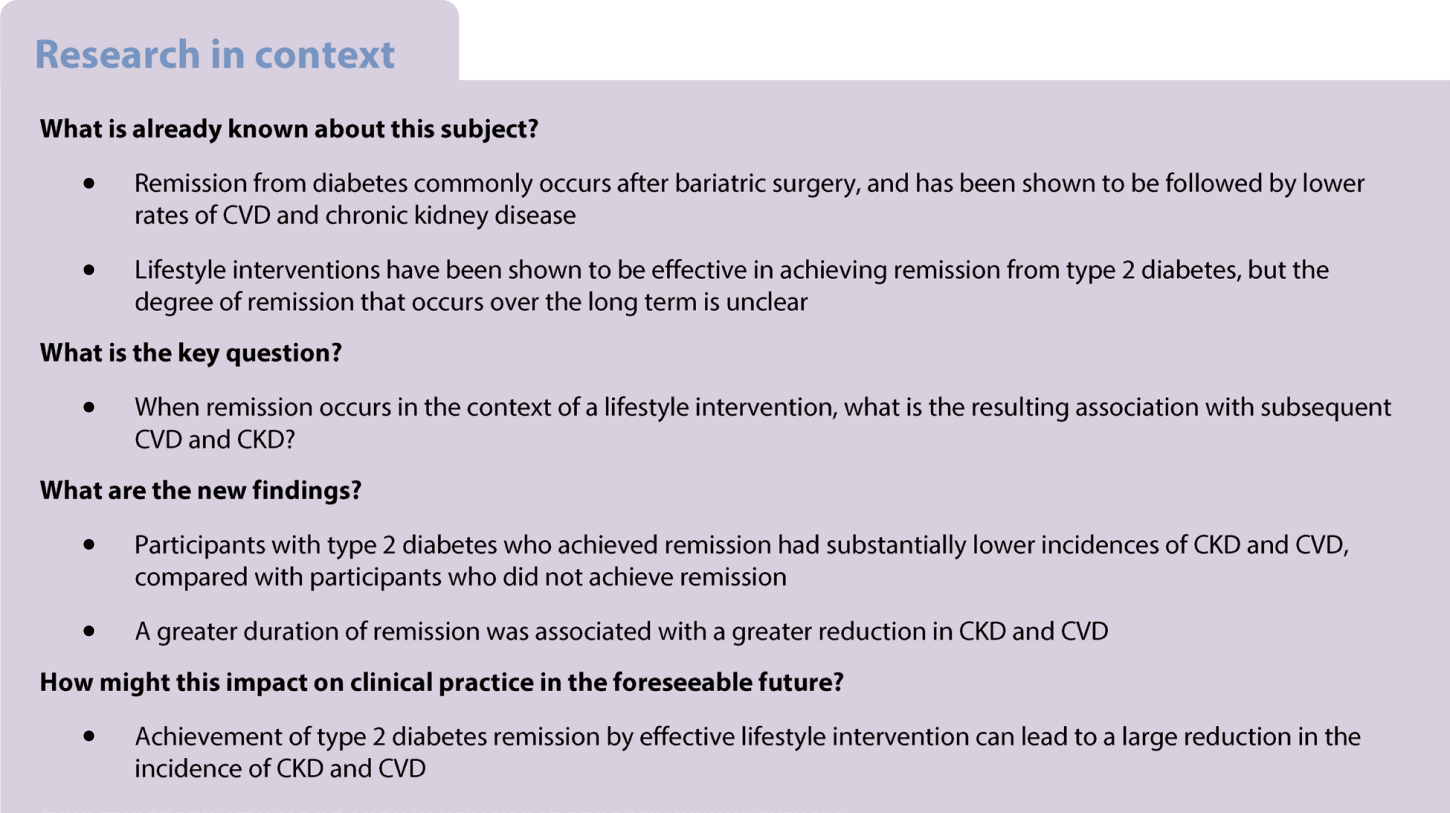


## Introduction

Remission from type 2 diabetes is increasingly seen as an attainable goal for many patients, with potential benefits in terms of long-term morbidity, quality of life and avoidance of further pancreatic beta cell failure [1–3]. Although remission from diabetes has usually been associated with bariatric surgery, lifestyle interventions have been shown to be effective in achieving remission from diabetes as well as prediabetes [4–8]. In the Look AHEAD study (ClinicalTrials.gov NCT00017953), 12% of all intervention participants, and 21% of those with fewer than 2 years of diabetes duration, achieved remission the first year, while 10% achieved 2 years of remission [4]. However, the prevalence of diabetes remission declined with each year of follow-up, such that by year 4, only 7% remained in remission. More recently, the cluster randomised DiRECT (Diabetes Remission Clinical Trial) trial in the UK implemented an even more intensive weight loss programme with low-carbohydrate meal replacements, and observed remission incidences of 46% and 36% over 1 and 2 years, respectively, among participants with diabetes of up to 6 years of duration [5, 8]. While the DiRECT trial is now being followed by a national evaluation to determine its viability as a primary care referral option for the UK National Health Service, understanding its long-term impact remains incomplete [9].

Remission from diabetes after bariatric surgery has been shown to be followed by lower incidences of CVD and chronic kidney disease (CKD) [7, 10–13]. However, bariatric surgery leads to substantially greater and longer-term normalisation of glucose than remission achieved through lifestyle intervention [7, 10–13]. Despite the growing interest in diabetes remission as a goal through lifestyle intervention, the long-term impact of achieving this goal remains unclear. Participants in the US Diabetes Prevention Program and the Da Qing Diabetes Prevention Follow-up Study who achieved reversal from prediabetes to normal glucose tolerance showed large improvements in CVD risk factors [14, 15]. Similarly, in an observational cohort of adults with diabetes in Southern England, participants who achieved remission had a 20–40% lower incidence of CVD and microvascular disease outcomes compared with participants who did not experience remission [16]. However, to our knowledge, no studies have yet examined the impact of diabetes remission in the context of lifestyle intervention on long-term health outcomes.

As the largest and longest trial of intensive weight loss ever conducted, Look AHEAD is a unique opportunity to study the long-term effects of remission on long-term metabolic control and risk of diabetes-related complications in the context of a long-term lifestyle intervention [17]. We conducted post hoc analyses of the Look AHEAD study to examine the incidence of apparent remission from type 2 diabetes in the overall study cohort and according to intervention status over a period of 12 years (median 10.2 years) from baseline. We examined whether diabetes remission in the context of a lifestyle intervention trial results in a reduction in the incidence of diabetes-related CKD and CVD.

## Methods

### Study design and study population

The Look AHEAD study was a multi-centre RCT that compared the effect of a 12 year intensive lifestyle intervention (ILI) with that of diabetes support and education (DSE) on CVD and other long-term health conditions [17, 18]. We conducted an observational post hoc analysis of participants in both groups, classified them based on remission status, and then compared long-term outcomes (described below) based on any remission, and the duration of remission, over a period of 12 years. These analyses were based on the core Look AHEAD dataset, with no additional data collected for these analyses.

The study recruited and randomised 5145 adults with overweight or obesity (BMI ≥25 kg/m^2^ for non-insulin users or BMI ≥27 kg/m^2^ for insulin users) aged 45–76 years with type 2 diabetes. Sex was classified based on self-report. For the purposes of study inclusion, diabetes was identified by self-reporting, with verification of at least one of the following: (1) participant self-report that glucose-lowering drugs are being taken to lower blood sugar; (2) written or verbal confirmation from the individual's physician that the participant has type 2 diabetes; (3) fasting plasma glucose value of at least 7.0 mmol/l confirmed on a subsequent day as per ADA criteria; or (4) a statement in the participant's medical record of type 2 diabetes or laboratory results that meet ADA criteria.

Exclusion criteria included an inability to walk two blocks, a lower limb amputation for non-traumatic causes, urine dipstick protein of 4+ (equivalent to approximately >1 g protein/day), serum creatinine > 124 μmol/l in women or > 133 μmol/l in men, or present treatment with dialysis, very poorly controlled HbA_1c_ (>11%), systolic BP >160 mmHg or diastolic BP >100) mmHg, triacylglycerol levels >6.8 mmol/l, or poor functional status, defined by an inability to complete a graded exercise test.

### Intervention

Details of the ILI have been provided previously [18, 19]. It included weekly group and individual sessions in the first 6 months, followed by two group sessions and one individual session per month for the second 6 months, and two contacts per month (at least one in person) for years 2–4. From years 4–12, participants were encouraged to attend monthly support sessions. The multi-component ILI aimed to reduce total caloric intake to 5021 to 7532 kJ/day (1200 to 1800 kcal/day) based on initial weight, reduce total fat and saturated fat intake to less than 30% and 10%, respectively, and increase physical activity to a level of 175 min per week using brisk walking and other moderate-intensity activities. Behavioural strategies, including self-monitoring and problem solving, were used to assist in meeting behavioural, weight, dietary and physical activity goals. Liquid meal replacements were provided in the first year to assist in meeting dietary goals.

Participants receiving DSE were offered three group sessions each year, focusing on diet, physical activity and social support, but individualised behavioural support was not provided. During periods of rapid weight loss, participants monitored blood sugar so that Look AHEAD medical staff could determine whether reductions in diabetes medications were needed to reduce risk of hypoglycaemia. However, medical or pharmacological care for control of hyperglycaemia, lipids and BP were provided by the participants’ physicians independently of the Look AHEAD study for both groups, as were any referrals or recommendations to undergo bariatric surgery.

### Assessments and outcomes

Participants attended a baseline clinic visit between August 2001 and April 2004, and annual follow-up visits for 4 years, followed by visits every 2 years thereafter for 12 years. At each visit, study personnel assessed the medications being taken, health status by questionnaire, body weight using a digital scale, height using a stadiometer, and HbA_1c_ via venous phlebotomy. Values for HbA_1c_, serum creatinine, and urinary albumin and creatinine were collected annually until year 4 and every 2 years thereafter. We defined remission of diabetes as a transition from meeting diabetes criteria to a prediabetes level (HbA_1c_ <48 mmol/mol, or 6.5%) with no use of glucose-lowering medications at each particular visit. As we did not perform a follow-up HbA_1c_ measurement within 3 months, as recommended by International Consensus, this definition should be considered an epidemiological surrogate definition of remission [1]. Further, we lacked data on the specific dates of cessation of medications or change in glucose levels, and thus quantified duration of remission in terms of the number of visits at which the participant met the remission definition. Thus, for a person with remission at one visit, the expected duration ranged from 0 to <1 year, and for a person with remission at two visits, the duration ranges from >1 to <3 years, while remission at three visits represents a range from >2 to <4 years.

We examined two primary health outcomes. The first outcome was the incidence of high-risk or very high-risk CKD based on the Kidney Disease Improving Global Outcomes (KDIGO) criteria, defined by (a) an eGFR less than 45 ml/min per 1.73 m^2^ regardless of the urine albumin to creatinine ratio; (b) eGFR less than 60 ml/min per 1.73 m^2^ and a urine albumin to creatinine ratio of at least 30 mg albumin per g creatinine; or (c) any eGFR level with a urine albumin to creatinine ratio greater than 300 mg albumin per g creatinine. The second outcome was the incidence of composite CVD using pre-specified primary outcomes (CVD death, non-fatal acute myocardial infarction, non-fatal stroke or admission for angina). These outcomes were selected because they represent long-term health impacts of remission through recognised causal (biological and treatment-related) pathways: renal disease is an indicator of the microvascular impact of extended high levels of glucose, and composite CVD is an indicator of the macrovascular impact. CKD incidence was based on data from the clinic visits, and data regarding CVD were determined through an adjudication process, as previously described [17, 20].

### Statistical analyses

Descriptive statistics were used to examine the yearly prevalence of any remission (HbA_1c_ <48 mmol/mol, or 6.5%, without use of glucose-lowering drugs) and the distribution of the number of visits at which this status was attained in the overall cohort and by intervention group. We used χ^2^ tests and ANOVA to compare demographic and health status characteristics and 1-year and 4-year changes in risk factors according to remission group. Based on inspection of the distribution of the number of visits at which any remission was achieved, we categorised participants into four groups: no remission, one visit with documented remission, two or three visits with remission and at least four visits with remission. For analyses stratified by intervention group, we further collapsed these into three groups (no remission, one visit with documented remission, two or more visits with remission) to allow sufficient precision of estimates. We calculated incidence rates as events divided by person-years. Our primary analyses used Cox proportional hazards regression to examine the hazard ratios between participants with no remission, remission at one visit, remission at two or three visits and remission at four visits, and adjusted for baseline age, BP, CVD history, HbA_1c_, years of diabetes and intervention status. Only remissions occurring prior to the events were considered in these analyses. Thus, if someone had an event prior to remission, the event would be counted and attributed to the group with no remission. Further follow-up data for remission from that point forward were not considered in the analysis of time until the first CKD/CVD event.

For additional perspective, we performed analyses in which the DSE group was treated as the referent group and their outcomes were compared with those of ILI participants with no remission, or one, two, three and >4 visits with documented remission. A two-sided *p* value < 0.05 was considered statistically significant.

## Results

Of the 5145 participants, we further excluded 296 (5.8%) who had already met our definition of remission at baseline, 107 (2.1%) with inadequate follow-up data to estimate remission over time, and 254 (4.9%) who had bariatric surgery over the course of the study, leaving an analytic sample size of 4488 individuals.

**Fig. 1:**
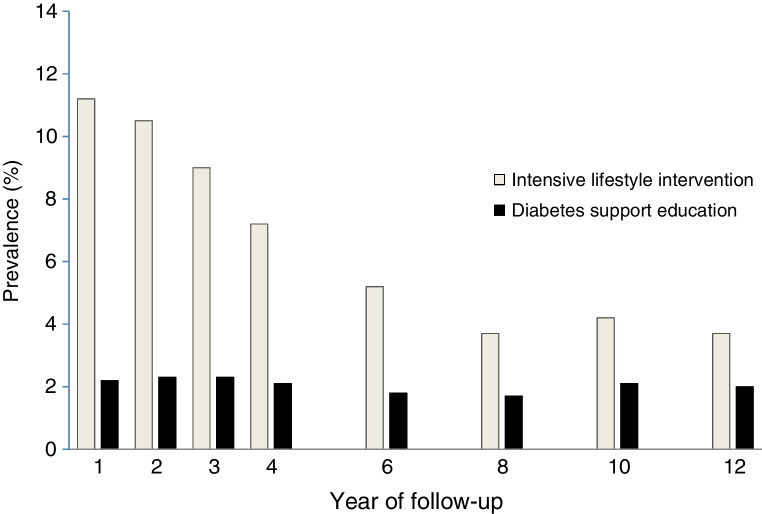
Prevalence of any remission by year of follow-up in the two Look AHEAD intervention groups

Among the analytic sample, 12.7% met our definition of remission for at least one follow-up visit. For the ILI group, the prevalence of remission was 11.2% at year 1 and declined about 0.7 percentage points every year, while that in the DSE group remained at approximately 2%, such that, by year 4, the prevalence of any remission was 3.5 times as high in the ILI group (7.2%) compared with the DSE group (2.1%), and, by year 12, the prevalence was approximately twice as high in the ILI group (3.7% vs 1.95%) (Fig. 1).

Remission status and remission duration were each significantly associated with baseline medication use, duration of disease and levels of HbA_1c_, fasting plasma glucose and systolic BP (Table 1). Participants who achieved remission at some point were more likely to have been taking no medications at baseline (19% to 38% across remission groups, vs 5% in those with no remission); participants who achieved remission for at least two visits were also less likely to have had prior CVD, and remission of any duration was associated with lower baseline levels of HbA_1c_, fasting glucose and systolic BP but not hyperlipidaemia. Longer periods of remission were also associated with shorter duration of diabetes, ranging from 6.0 to 2.0 years of diabetes across those with no remission to those with remission for at least four visits.

**Table 1:** Characteristics of participants at baseline, and post-baseline weight and risk factor changes, according to remission status

	Remission status (number of visits)				*p* value
	No remission	One visit	Two or three visits	Four or more visits	
Number of participants	3919	242	182	145	
Proportion in ILI group	46.5	60.7	73.6	84.1	
Age (years)	58.9 (6.7)	59.5 (6.9)	59.6 (6.7)	59.1 (7.0)	0.29
Male	41.6	41.3	42.9	50.3	0.21
Race/ethnicity (non-white)	38.2	40.9	34.6	30.3	0.14
Education (years)					
<13	20.6	19.5	18.7	20.1	0.29
13 to <16	37.7	34.0	35.2	29.2	
16+	39.6	42.7	44.0	48.6	
Other	2.1	3.7	2.2	2.1	
Current smoking	4.7	4.1	3.3	2.8	0.57
History of CVD	14.8	16.5	5.5	11.0	0.002
Diabetes mellitus medications					
None	4.6	18.5	29.9	38.2	<0.0001
Oral medications alone	74.1	71.2	63.3	59.7	
Any insulin	21.2	10.3	6.8	2.1	
Diabetes duration (years)	6.0 (3.0–10)	3.0 (1.0–7.0)	3.0 (1.0–6.0)	2.0 (1.0–4.0)	<0.0001
BMI (kg/m^2^)	35.8 (5.7)	35.5 (5.7)	34.8 (6.0)	35.3 (5.4)	0.12
HbA_1c_					
mmol/mol	57.7 (12.7)	52.8 (10.3)	50.5 (8.7)	48.2 (8.2)	<0.0001
%	7.4 (1.2)	7.0 (0.9)	6.8 (0.8)	6.6 (0.7)	<0.0001
Fasting glucose (mmol/l)	8.7 (2.6)	8.2 (2.0)	7.7 (1.7)	7.3 (1.6)	<0.0001
Systolic BP (mmHg)	129.2 (17.1)	129.1 (18.1)	127.3 (15.7)	125.6 (16.2)	0.048
LDL-cholesterol (mmol/l)	2.9 (0.8)	2.9 (0.9)	2.9 (0.8)	2.8 (0.8)	0.5045
HDL-cholesterol (mmol/l)	1.1 (0.3)	1.1 (0.3)	1.1 (0.3)	1.1 (0.3)	0.5166
Post-baseline risk factor changes from 0 to 1 year					
HbA_1c_					
mmol/mol	−27.3 (11.6)	−31.0 (9.8)	−31.4 (8.9)	−31.7 (8.3)	<0.0001
%	−0.4 (1.1)	−0.7 (0.9)	−0.7 (0.8)	−0.7 (0.8)	<0.0001
Weight (kg)	−4.0 (6.5)	−7.3 (7.3)	−9.8 (8.2)	−12.3 (8.3)	<0.0001
Systolic BP (mm/Hg)	−4.6 (17.1)	−5.0	−7.3 (17.7)	−7.7 (16.2)	0.03
LDL-cholesterol (mmol/l)	-0.15 (0.76)	-0.13 (0.68)	−0.05 (0.64)	−0.08 (0.68)	0.2051
HDL-cholesterol (mmol/l)	0.06 (0.17)	0.09 (0.21)	0.10 (0.19)	0.13 (0.20)	<0.0001
Fitness (METs)	11.3 (24.8)	20.6 (35.0)	24.7 (31.3)	30.0 (34.8)	<0.0001
Post-baseline risk factor changes from 0 to 4 years					
HbA_1c_					
mmol/mol	−24.5 (15.3)	−26.0 (12.2)	−27.8 (12.0)	−31.9 (8.6)	<0.0001
%	−0.4 (1.1)	−0.7 (0.9)	−0.7 (0.8)	−0.7 (0.8)	<0.0001
Weight (kg)	−2.2 (7.2)	−4.5 (7.7)	−6.2 (8.1)	−9.6 (8.9)	<0.0001
Systolic BP (mm/Hg)	−3.9 (19.1)	−4.6 (18.9)	−1.5 (20.5)	−5.9 (18.0)	0.21
LDL-cholesterol (mmol/l)	−0.48 (0.89)	−0.44 (0.86)	−0.37 (0.89)	−0.26 (0.86)	0.0139
HDL-cholesterol (mmol/l)	0.08 (0.21)	0.08 (0.21)	0.11 (0.21)	0.16 (0.23)	<0.0001
Fitness (METs)	0.6 (25.1)	2.3 (25.6)	9.0 (28.9)	14.6 (35.6)	<0.0001

Data are means (SD), median (range) or %

MET, metabolic equivalent

### Risk factor changes by remission status

Remission was also significantly associated with the changes in weight and risk factors over years 0 to 4 (Table 1). Whereas the mean weight loss after 1 and 4 years was 4 and 2.2 kg, respectively, for participants without remission, those with remission for at least one visit had lost 7.3 and 4.5 kg of body weight at years 1 and 4, respectively, and those with remission for at least four visits had lost 12.3 and 9.6 kg of body weight. There were also significantly greater improvements in HDL-cholesterol and fitness after 1 and 4 years, and significantly greater systolic BP improvements after 1 year among participants with remission compared with those without remission. Systolic BP decreased more and HDL-cholesterol increased more among participants who achieved a greater duration of diabetes remission. In contrast to the other risk factors, a smaller reduction in LDL-cholesterol was observed for those who achieved remission. In analyses stratified by intervention condition, we observed similar associations of remission status with baseline risk factors and short-term changes, except that the association of weight loss with duration of remission was stronger among ILI participants than among DSE participants (see electronic supplementary material [ESM] Table 1).

### Primary outcomes

**Fig. 2:**
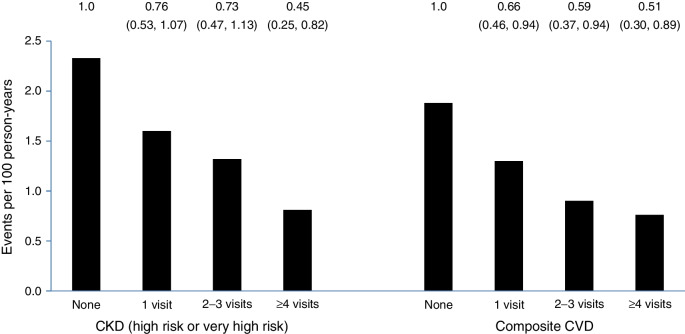
Incidence of CKD and CVD based on number of visits with remission. The HR and 95% CI values are shown at the top

Compared with participants who did not achieve remission, participants who experienced any remission had a 33% lower rate of CKD (HR 0.67; 95% CI 0.52, 0.87) and a 40% lower rate of CVD (HR 0.60; 95% CI 0.47, 0.79) in multivariate analyses (Table 2 and Fig. 2). This association had an underlying dose–response relationship, with a notable difference between those who had no remission and any remission, and the rates of CKD and CVD each being lowest among participants who had remission for at least four visits (HR 0.45; 95% CI 0.25, 0.82 for CKD; HR 0.51; 95% CI 0.30, 0.89 for CVD).

**Table 2:** Incidence rates and hazard ratios for primary outcomes (CKD and composite CVD) according to diabetes remission status among the overall cohort of Look AHEAD participants

	Remission status (number of visits)				
	No remission	One visit	Two or three visits	Four or more visits	Any
High-risk/very high-risk CKD					
Number of events	769/3628	33/215	21/159	11/130	65/504
Rate (events/100 person-years)	2.33 (2.17, 2.50)	1.60 (1.14, 2.25)	1.32 (0.86, 2.03)	0.81 (0.45, 1.46)	1.30 (1.02, 1.65)
Crude HR	1.0	0.68 (0.48, 0.96)	0.56 (0.36, 0.87)	0.33 (0.18, 0.60)	0.54 (0.42, 0.70)
Multivariate HR^a^	1.0	0.76 (0.53, 1.07)	0.73 (0.47, 1.13)	0.45 (0.25, 0.82)	0.67 (0.52, 0.87)
Composite CVD^b^					
Events	785/3863	33/223	19/176	13/140	65/539
Rate (events/100 person-years)	1.88 (1.75, 2.02)	1.30 (0.92, 1.82)	0.90 (0.57, 1.40)	0.76 (0.44, 1.31)	1.02 (0.80, 1.30)
Crude HR	1.0	0.69 (0.48, 0.97)	0.47 (0.30, 0.74)	0.40 (0.23, 0.69)	0.54 (0.42, 0.69)
Multivariate HR^a^	1.0	0.66 (0.46, 0.94)	0.59 (0.37, 0.94)	0.51 (0.30, 0.89)	0.60 (0.47, 0.79)

^a^Multivariate models adjusted for baseline mean BP, CVD history, diabetes duration, HbA_1c_ and intervention arm

^b^Incidence of composite CVD using the pre-specified primary outcomes (CVD death, non-fatal acute myocardial infarction, non-fatal stroke or admission for angina)

In analyses treating DSE as the referent group (irrespective of remission status), participants in the ILI group with any remission had a 40% lower rate of CKD (HR 0.60; 95% CI 0.44, 0.82) and a 23% lower rate of CVD (HR 0.77; 95% CI 0.58, 1.03) (ESM Table 2). Participants in the ILI group with remission for at least four visits had a 60% lower rate of CKD (HR 0.40; 95% CI 0.21, 0.78) and a 35% lower rate of CVD (HR 0.65; 95% CI 0.37, 1.13).

Tests of interactions between study arm by remission status were not significant for CKD (*p*=0.50 and *p*=0.43 in crude and multivariate analyses, respectively), but were significant for CVD (*p*=0.04 and *p*=0.02 for crude and multivariate analyses, respectively). In multivariate analyses stratified by randomisation group, remission was associated with a 34% reduction in CKD (HR 0.66; 95% CI 0.48, 0.91) but not CVD (HR 0.78; 95% CI 0.58, 1.05) (Table 3) among those assigned to the ILI group. Among those in the DSE group, remission was significantly associated with a 68% reduction in CVD (HR 0.32; 95% CI 0.18, 0.59) but was not associated with a reduction in CKD (HR 0.70; 95% CI 0.45, 1.10).

**Table 3:** Primary outcomes for the two intervention groups according to remission status

	Remission status			
	DSE group		ILI group	
	No remission	Any remission	No remission	Any remission
High-risk/very high-risk CKD				
Events	426/1944	20/139	343/1684	45/365
Rate (events/100 person-years)	2.42 (2.20, 2.66)	1.46 (0.94, 2.26)	2.22 (2.00, 2.47)	1.24 (0.92, 1.65)
Crude HR	1.0	0.60 (0.38, 0.94)	1.0	0.53 (0.39, 0.72)
Multivariate HR	1.0	0.70 (0.45, 1.10)	1.0	0.66 (0.48, 0.91)
Composite CVD^a^				
Events	412/2058	11/155	373/1805	54/384
Rate (events/100 person-years)	1.86 (1.69, 2.05)	0.60 (0.33, 1.08)	1.91 (1.72, 2.11)	1.19 (1.91, 1.55)
Crude HR	1.0	0.32 (0.17, 0.58)	1.0	0.62 (0.47, 0.83)
Multivariate HR	1.0	0.32 (0.18, 0.59)	1.0	0.78 (0.58, 1.05)

^a^Incidence of composite CVD using the pre-specified primary outcomes (CVD death, non-fatal acute myocardial infarction, non-fatal stroke or admission for angina)

## Discussion

In this long-term follow-up of participants in the Look AHEAD study, we observed three main findings related to the implications of achieving diabetes remission. First, although 11% of intervention participants achieved remission at year 1 of follow-up, the percentage of participants with remission had decreased to 4% by the 8th year of the study. Second, despite the relatively short-lived durations of most episodes of remission, we found that any achievement of remission was associated with 33% and 40% lower rates of CKD and CVD, respectively, compared with participants who did not achieve remission, and risk reduction was even greater (55% and 49%, respectively) among those who had evidence of at least 4 years of remission. Third, participants with a short duration of diabetes, low starting HbA_1c_ and a large magnitude of weight loss were most likely to experience remission.

Remission from type 2 diabetes may be associated with lower rates of CKD, CVD and other long-term health outcomes through several pathways. First, the sustained reduction in HbA_1c_ and insulin resistance may benefit the vascular endothelium, microcirculation and reduce atherosclerosis progression [21]. Second, remission benefits may occur through the diverse physiological effects of extensive weight loss, including reductions in hyperglycaemia, BP, insulin resistance, inflammation and hepatic fat levels [2, 22]. In these analyses, participants with any remission had a slightly greater net weight loss than those without remission (2%), but those with extended remission had an 8% greater weight loss. Previous post hoc cohort analyses of the Look AHEAD study found that participants who met the weight loss goal of 10% at 1 year had a 20% lower incidence of the primary CVD outcome despite the overall null finding of the Look AHEAD intervention [17, 23]. Third, the behavioural changes associated with greater weight loss and intervention adherence, including better dietary quality, increased physical activity and higher attained physical fitness, may drive further health benefits. Finally, the achievement of remission may be a marker for other unmeasured differences or advantages arising from care and risk factor management. The observational study design limits inferences about which of these mechanisms of action account for the long-term benefit.

Compared with our study, the DiRECT study and the DIADEM-I study (Diabetes Intervention Accentuating Diet and Enhancing Metabolism) found considerably greater remission and generally similar weight loss over the first 2 years [4, 5, 24]. The DiRECT study found remission rates of 46% and 36% among intervention participants at 1 and 2 years, respectively (compared with 4% and 3% of control participants), accompanying 9.5% and 5.3% net weight losses, respectively, after 1 and 2 years [5, 25]. The DIADEM-I study reported a net 1-year remission rate of 61% (compared with 12% of control participants) and a 6.1% net weight loss [24]. By comparison, the Look AHEAD study reported 12% and 10% remission rates years 1 and 2, respectively, and a 1-year net weight loss of 8%. However, the DiRECT and DIADEM-I studies differed from the Look AHEAD study in other ways. Both studies employed total diet replacement, with targets of approximately 800–850 kcal/day, and importantly, employed an active protocol-driven approach to medication removal. After 6 months, the DiRECT study re-introduced food [5] and provided advice to increase physical activity during the maintenance period [26]. Several other studies have observed similarly profound effects on HbA_1c_ with dietary replacement and intensive weight loss over a shorter time period than the DiRECT and Look AHEAD Studies [27–30]. In contrast, the Look AHEAD study aimed for diets of 1200–800 kcal to achieve long-term weight loss, and passively relied upon primary care providers to remove medications as appropriate [19]. The Look AHEAD study was also distinct in terms of its relatively strong emphasis on physical activity, the longer duration of diabetes at enrolment of participants (mean 6 years), and the inclusion of people with prior coronary heart disease. We are aware of only one study examining long-term outcomes following remission in non-surgical settings: a 7-year follow-up of a cohort of adults with diabetes in Southern England that found a 20–40% lower incidence of CVD and microvascular disease outcomes among participants who experienced diabetes remission, but did not collect information on intervention participation [16].

Collectively, the results of these trials suggest that remission in people with recently diagnosed type 2 diabetes is achievable through lifestyle intervention resulting in substantial weight loss, but that its effectiveness may depend upon a strong response to interventions in selected subgroups of participants. Previous analyses of Look AHEAD data showed that the percentage of participants achieving remission was twice as great in participants with a 1-year weight loss >6.5% and <2 years diabetes duration, and about 50% greater among participants with HbA_1c_ <48 mmol/mol (6.5%) or in the top tertile of fitness change [4]. In the DiRECT study, duration of diabetes did not predict remission probability within the trial, but the average duration was only 3 years and all participants had a duration of fewer than 6 years [5, 8, 31]. Further, in post hoc analyses, weight loss and programme attendance were the strongest predictors of remission [31].

Observational studies have shown that rates of diabetes remission in the community in the absence of intensive interventions are particularly low [32–34]. In the UK National Diabetes Audit, which did not assess intentional weight loss, remission was only 1% in the overall population with diabetes and 3% in those with a recent diagnosis, and 8% among the subset with a large weight loss [34]. Achievement of and duration of remission were associated with several differences at baseline, including shorter duration of diabetes, higher levels of education, and lower starting HbA_1c_, fasting glucose levels and systolic BP. Our analyses adjusted for these factors and thus probably did not confound the association of remission with long-term outcomes. However, these differences may indicate that people who are earlier in the natural history of diabetes are more likely to achieve and benefit from attempts at remission. Our observation of higher LDL-cholesterol levels in participants achieving remission is surprising, but may be a reflection of the higher statin use among participants in the DSE group, as observed in the primary trial.

There are several limitations to these analyses. First, we lacked the necessary data to directly replicate the newly recommended clinical definition of remission, and instead relied upon an epidemiological definition as a proxy [1]. Second, we lacked data to pinpoint the onset of diabetes remission with precision, and instead used the number of visits with remission as a proxy for time without remission, thus underestimating the time in remission for some and overestimating it for others, and increasing the error variance in the estimates of risk reduction. Third, our analyses excluded approximately 10% of the overall sample due to the participants having bariatric surgery, inadequate data or already meeting the remission definition at baseline. Fourth, because the study did not have a randomised design, it is also possible that participants who achieved remission sought more intensive care and risk factor management, that drove the reduction in outcomes. Thus, there may be additional unmeasured differences in the characteristics of participants who achieved remission that also correlate with better health outcomes. Fifth, we lacked the power to stratify our core analyses by intervention status. Remission also occurred in DSE participants, albeit less frequently (7%, compared with vs 22% in ILI participants), and accounted for about one third of those with remission. Finally, we lacked the necessary data to determine which factors explained the association of remission with reductions in the incidence of CKD and CVD, including whether sex played a role or whether findings varied by sex. We speculate that remission in the DSE group was driven by similar lifestyle and risk factor changes to the factors driving remission in the intervention group, but our study was not adequately powered to examine predictors of remission within the DSE group, as only 11 CVD events and 20 CKD events occurred in DSE participants with diabetes remission. Finally, although the magnitude of weight loss appears to be a key driver in the reduction in CKD and CVD risk, whether targeting remission to influence subsequent CKD and CVD risk is more effective than targeting optimal management of the risk factors themselves cannot be tested by these analyses.

Despite the promising efficacy and outcomes of lifestyle-based remission in this and other studies, the viability of focusing on remission with lifestyle interventions as a major priority for clinical and public health efforts remains undetermined. On the one hand, these findings may drive a paradigm shift whereby selected subsets of the population are actively encouraged to strive beyond prevention of diabetes and its complications, to regression in risk status, in order to optimise long-term health outcomes [14, 15]. On the other hand, the long-term sustainability of such intensive interventions is unclear, and their incremental benefit above and beyond what may be achieved by targeting optimal risk factor management and more modest weight loss has not been tested in experimental settings. These questions underscore the need for continued follow-up in remission studies, as well as rigorous evaluation of real-world programmes of remission as they develop in the future.

### Supplementary Information

Below is the link to the electronic supplementary material.

Supplementary file1 (PDF 261 KB)

## Data Availability

Look AHEAD data are available on request from the National Institute of Diabetes and Digestive and Kidney Diseases (NIDDK) data repository (https://repository.niddk.nih.gov/studies/look-ahead/).

## Abbreviations

CKD: Chronic kidney disease
DSE: Diabetes support and education
ILI: Intensive lifestyle intervention
