# Supplementary material for: Impact of remission from type 2 diabetes on long-term health outcomes: findings from the Look AHEAD study
Source: Diabetologia. 2024 Jan 18;67(3):459–69. doi: 10.1007/s00125-023-06048-6 (PMC10844408; doi:10.1007/s00125-023-06048-6)
Supplement: Supplementary file 1 — Supplementary file1 (PDF 261 KB) [file 125_2023_6048_MOESM1_ESM.pdf]

ESSENTIAL SUPPLEMENTARY MATERIAL:

ESM Table 1. Characteristics of participants and post-baseline weight and risk factor changes, according to remission status and intervention status.

	DSE				ILI			
**Characteristics**	**No remission**	**1 remission**	**≥ 2 remissions**	**p value**	**No remission**	**1 remission**	**≥ 2 remissions**	**p value**
Number	2097	95	71		1822	147	256	
Baseline								
Age (mean years)	59.0 (6.8)	60.6 (7.0)	59.4 (7.5)	0.09	58.7 (6.7)	58.7 (6.8)	59.4 (6.6)	0.28
Male (%)	884 (42.2)	37 (38.9)	23 (32.4)	0.22	747 (41.0)	63 (42.9)	128 (50.0)	0.02
(% non-white)	794 (37.9)	38 (40.0)	27 (38.0)	0.91	704 (38.7)	61 (41.5)	80 (31.3)	0.05
Educ: < 13 years (%)	428 (20.4)	22 (23.2)	12 (16.9)	0.46	380 (20.9)	25 (17.1)	51 (20.0)	0.07
13 to < 16 years (%)	797 (38.0)	34 (35.8)	20 (28.2)	0.46	681 (37.4)	48 (32.9)	86 (33.7)	0.07
16+ y (%)	819 (39.1)	37 (38.9)	37 (52.1)	0.46	731 (40.1)	66 (45.2)	113 (44.3)	0.07
Other	53 (2.5)	2 (2.1)	2 (2.8)	0.46	29 (1.6)	7 (4.8)	5 (2.0)	0.07
smoking (%)	89 (4.3)	5 (5.3)	2 (2.8)	0.74	95 (5.2)	5 (3.4)	8 (3.1)	0.24
History CVD (%)	294 (14.0)	17 (17.9)	5 (7.0)	0.13	286 (15.7)	23 (15.6)	21 (8.2)	0.007
DM meds: none	124 (6.1)	20 (21.7)	26 (37.1)	<.0001	52 (2.9)	23 (16.3)	82 (32.7)	<0.0001
Oral meds alone	1488 (73.6)	60 (65.2)	38 (54.3)	<.0001	1324 (74.7)	106 (75.2)	160 (63.7)	<0.0001
Any insulin use	410 (20.3)	12 (13.0)	6 (8.6)	<.0001	396 (22.3)	12 (8.5)	9 (3.6)	<0.0001
DM duration (med years)	5.0 (3.0-10.0)	3.5 (1.0-7.0)	2.0 (1.0-5.0)	<.0001	6.0 (3.0-11.0)	3.0 (2.0-6.0)	2.0 (1.0-5.0)	<0.0001
BMI (mean)	35.7 (5.6)	36.4 (5.7)	36.2 (6.2)	0.44	35.8 (5.9)	34.9 (5.6)	34.7 (5.5)	0.005
HbA1c (mean mmol/mol)	57.5 (12.8)	51.8 (10.4)	48.1 (8.0)	<.0001	57.8 (12.6)	53.4 (10.2)	49.9 (8.7)	<0.0001
(mean %, SD)	7.4 (1.2)	6.9 (1.0)	6.5 (0.7)	<.0001	7.4 (1.2)	7.0 (0.9)	6.7 (0.8)	<0.0001
Fasting plasma glucose (mean mmol/l)	8.7 (2.6)	8.0 (2.2)	7.3 (1.4)	<.0001	8.7 (2.6)	8.4 (1.9)	7.6 (1.7)	<0.0001
**Post-Baseline Risk Factor Changes**								
**Year 1 change**								
Weight (kg)	-0.6 (4.4)	-2.2 (5.0)	-2.1 (6.5)	<.0001	-7.8 (6.3)	-10.7 (6.5)	-13.4 (7.0)	<0.0001
HbA1c (mean mmol/mol)	-24.7 (11.1)	-28.1 (9.5)	-27.3 (8.0)	0.0021	-30.3 (11.5)	-32.9 (9.5)	-32.7 (8.5)	0.0004
(mean %, SD)	-0.1 (1.0)	-0.4 (0.9)	-0.3 (0.7)	0.0021	-0.6 (1.1)	-0.9 (0.9)	-0.8 (0.8)	0.0004
SBP (mean mm/Hg)	-2.8 (16.7)	-2.1 (16.3)	-3.0 (18.0)	0.9111	-6.5 (17.2)	-6.9 (18.6)	-8.7 (16.5)	0.1625
LDL cholesterol (mean mmol/l)	-1.7 (8.5)	-2.7 (8.2)	-1.3 (10.0)	0.5231	-2.9 (8.6)	-3.0 (8.6)	-3.2 (8.2)	0.9179
HDL cholesterol (mean mmol/l)	0.0 (0.2)	0.1 (0.2)	0.0 (0.2)	0.5013	0.1 (0.2)	0.1 (0.2)	0.1 (0.2)	<0.0001
Fitness (METs)	4.7 (20.6)	8.1 (25.0)	14.1 (32.1)	0.0012	18.4 (27.0)	28.2 (38.0)	30.6 (32.4)	<0.0001
**Year 4 change**								
Weight (kg)	-0.5 (6.9)	-2.4 (7.6)	-3.9 (9.5)	<.0001	-4.0 (7.1)	-5.9 (7.4)	-8.8 (8.1)	<0.0001
HbA1c (mean mmol/mol)	-24.0 (15.8)	-26.2 (13.5)	-28.6 (9.2)	0.0318	-25.1 (14.8)	-25.9 (11.4)	-30.0 (11.1)	<0.0001

	DSE				ILI			
**Characteristics**	**No remission**	**1 remission**	**≥ 2 remissions**	**p value**	**No remission**	**1 remission**	**≥ 2 remissions**	**p value**
(mean %, SD)	-0.0 (1.4)	-0.2 (1.2)	-0.5 (0.8)	0.0318	-0.1 (1.3)	-0.2 (1.0)	-0.6 (1.0)	<0.0001
SBP (mean mm/Hg)	-3.6 (19.1)	-5.0 (20.8)	-3.4 (19.8)	0.8035	-4.2 (19.1)	-4.3 (17.6)	-3.6 (19.4)	0.8763
LDL cholesterol (mean mmol/l)	-0.5 (0.9)	-0.4 (0.8)	-0.2 (0.8)	0.0181	-0.4 (0.9)	-0.5 (0.9)	-0.3 (0.9)	0.1876
HDLcholesterol (mean mmol/l)	0.1 (0.2)	0.0 (0.2)	0.1 (0.2)	0.6973	0.1 (0.2)	0.1 (0.2)	0.1 (0.2)	0.0012
Fitness (METs)	-2.1 (23.7)	-4.4 (21.8)	9.4 (35.1)	0.0009	3.6 (26.3)	6.1 (26.8)	12.2 (31.5)	<.0001

ESM Table 2. Incidence rates and hazard ratios for primary outcomes (chronic kidney disease and composite CVD) according to diabetes remission status comparing DSE participants to ILI participants, classified according to achievement of remission

	**Remission Status (Number of visits)**					
**Baseline**	**DSE**	**ILI with No Remission**	**ILI with Remission for 1 visit**	**ILI with Remission for 2-3 visits**	**ILI with Remission for 4+ visits**	**ILI with Any Remission**
**High / Very High Risk CKD**						
Events	446	343	18	18	9	45
Rate (events / 100 person years)	2.35 (2.14, 2.58)	2.22 (2.00, 2.47)	1.38 (0.87, 2.19)	1.53 (0.96, 2.42)	0.78 (0.40, 1.49)	1.24 (0.92, 1.65)
Crude HR	1.0	0.96 (0.83, 1.11)	0.58 (0.36, 0.93)	0.64 (0.40, 1.02)	0.32 (0.17, 0.62)	0.52 (0.38, 0.70)
Multivariate HR**	1.0	0.92 (0.80, 1.06)	0.62 (0.39, 1.00)	0.78 (0.48, 1.25)	0.40 (0.21, 0.78)	0.60 (0.44, 0.82)
**Composite CVD***						
Events	423	373	24	17	13	54
Rate (events / 100 person years)	1.76 (1.60, 1.94)	1.91 (1.72, 2.11)	1.53 (1.02, 2.28)	1.12 (0.70, 1.81)	0.89 (0.52, 1.54)	1.19 (0.91, 1.55)
Crude HR	1.0	1.08 (0.94, 1.24)	0.87 (0.57, 1.31)	0.63 (0.39, 1.03)	0.51 (0.29, 0.88)	0.67 (0.51, 0.89)
Multivariate HR**	1.0	1.04 (0.90, 1.19)	0.85 (0.56, 1.30)	0.78 (0.47, 1.29)	0.65 (0.37, 1.13)	0.77 (0.58, 1.03)

*Incidence of composite CVD using the pre-specified primary outcome (CVD death, non-fatal acute MI, non-fatal stroke or admission for angina; **multivariate models adjusting for baseline mean blood pressure, CVD history, diabetes duration, HbA1c; and intervention arm.

Look AHEAD Research Group at End of Intervention

Clinical Sites

The Johns Hopkins University Frederick L. Brancati, MD, MHS[1]; Jeanne M. Clark, MD, MPH[1] (Co-Principal Investigators); Lee Swartz[2]; Jeanne Charleston, RN[3]; Lawrence Cheskin, MD[3]; Kerry Stewart, EdD[3]; Richard Rubin, PhD[3]; Jean Arceci, RN; Susanne Danus; David Bolen; Danielle Diggins; Sara Evans; Mia Johnson; Joyce Lambert; Sarah Longenecker; Kathy Michalski, RD; Dawn Jiggetts; Chanchai Sapun; Maria Sowers; Kathy Tyler

Pennington Biomedical Research Center George A. Bray, MD[1]; Allison Strate, RN[2]; Frank L. Greenway, MD[3]; Donna H. Ryan, MD[3]; Donald Williamson, PhD[3]; Timothy Church, MD[3] ; Catherine Champagne, PhD, RD; Valerie Myers, PhD; Jennifer Arceneaux, RN; Kristi Rau; Michelle Begnaud, LDN, RD, CDE; Barbara Cerniauskas, LDN, RD, CDE; Crystal Duncan, LPN; Helen Guay, LDN, LPC, RD; Carolyn Johnson, LPN, Lisa Jones; Kim Landry; Missy Lingle; Jennifer Perault; Cindy Puckett; Marisa Smith; Lauren Cox; Monica Lockett, LPN

The University of Alabama at Birmingham Cora E. Lewis, MD, MSPH[1]; Sheikilya Thomas, MPH[2]; Monika Safford, MD[3]; Stephen Glasser, MD[3]; Vicki DiLillo, PhD[3]; Charlotte Bragg, MS, RD, LD; Amy Dobelstein; Sara Hannum, MA; Anne Hubbell, MS; Jane King, MLT; DeLavallade Lee; Andre Morgan; L. Christie Oden; Janet Wallace, MS; Cathy Roche, PhD,RN, BSN; Jackie Roche; Janet Turman

Harvard Center

Massachusetts General Hospital. David M. Nathan, MD[1]; Enrico Cagliero, MD[3]; Kathryn Hayward, MD[3]; Heather Turgeon, RN, BS, CDE[2]; Valerie Goldman, MS, RD[2]; Linda Delahanty, MS, RD[3]; Ellen Anderson, MS, RD[3]; Laurie Bissett, MS, RD; Virginia Harlan, MSW; Theresa Michel, DPT, DSc, CCS; Mary Larkin, RN; Christine Stevens, RN

Joslin Diabetes Center: Edward S. Horton, MD[1]; Sharon D. Jackson, MS, RD, CDE[2]; Osama Hamdy, MD, PhD[3]; A. Enrique Caballero, MD[3]; Sarah Bain, BS;
Elizabeth Bovaird, BSN, RN; Barbara Fargnoli, MS,RD; Jeanne Spellman, BS, RD; Kari Galuski, RN; Ann Goebel-Fabbri, PhD; Lori Lambert, MS, RD; Sarah Ledbury, MEd, RD; Maureen Malloy, BS; Kerry Ovalle, MS, RCEP, CDE

Beth Israel Deaconess Medical Center: George Blackburn, MD, PhD[1]; Christos Mantzoros, MD, DSc[3]; Ann McNamara, RN; Kristina Spellman, RD

University of Colorado Anschutz Medical Campus James O. Hill, PhD[1]; Marsha Miller, MS RD[2]; Holly Wyatt, MD[3] , Brent Van Dorsten, PhD[3]; Judith Regensteiner, PhD[3]; Debbie Bochert; Ligia Coelho, BS; Paulette Cohrs, RN, BSN; Susan Green; April Hamilton, BS, CCRC; Jere Hamilton, BA; Eugene Leshchinskiy; Loretta Rome, TRS; Terra Thompson, BA; Kirstie Craul, RD,CDE; Cecilia Wang, MD

Baylor College of Medicine John P. Foreyt, PhD[1]; Rebecca S. Reeves, DrPH, RD[2]; Molly Gee, MEd, RD[2]; Henry Pownall, PhD[3]; Ashok Balasubramanyam, MBBS[3]; Chu-Huang Chen, MD, PhD[3]; Peter Jones, MD[3]; Michele Burrington, RD, RN; Allyson Clark Gardner,MS, RD; Sharon Griggs; Michelle Hamilton; Veronica Holley; Sarah Lee; Sarah Lane Liscum, RN, MPH; Susan Cantu-Lumbreras; Julieta Palencia, RN; Jennifer Schmidt; Jayne Thomas, RD; Carolyn White

The University of Tennessee Health Science Center

University of Tennessee East. Karen C. Johnson, MD, MPH[1]; Carolyn Gresham, RN[2]; Mace Coday, PhD; Lisa Jones, RN; Lynne Lichtermann, RN, BSN; J. Lee Taylor, MEd, MBA; Beate Griffin, RN; Donna Valenski

University of Tennessee Downtown. Abbas E. Kitabchi, PhD, MD[1]; Ebenezer Nyenwe, MD[3]; Helen Lambeth, RN, BSN[2]; Moana Mosby, RN; Amy Brewer, MS, RD,LDN; Debra Clark, LPN; Andrea Crisler, MT; Gracie Cunningham; Debra Force, MS, RD, LDN; Donna Green, RN; Robert Kores, PhD; Renate Rosenthal, PhD; Elizabeth Smith, MS, RD, LDN

University of Minnesota Robert W. Jeffery, PhD[1]; Tricia Skarphol, MA[2]; Carolyn Thorson, CCRP[2]; John P. Bantle, MD[3]; J. Bruce Redmon, MD[3]; Richard S. Crow, MD[3]; Kerrin Brelje, MPH, RD; Carolyne Campbell; Lisa Hoelscher, MPH, RD, CHES; Melanie Jaeb, MPH, RD; LaDonna James; Patti Laqua, BS, RD; Vicki A. Maddy, BS, RD; Therese Ockenden, RN; Birgitta I. Rice, MS, RPh, CHES; Ann D. Tucker, BA; Mary Susan Voeller, BA; Cara Walcheck, BS,RD

St. Luke's Roosevelt Hospital Center Xavier Pi-Sunyer, MD[1]; Jennifer Patricio, MS[2]; Carmen Pal, MD[3]; Lynn Allen, MD;Janet Crane, MA, RD, CDN; Lolline Chong, BS, RD; Diane Hirsch, RNC, MS, CDE; Mary Anne Holowaty, MS, CN; Michelle Horowitz, MS, RD

University of Pennsylvania Thomas A. Wadden, PhD [1]; Barbara J. Maschak-Carey, MSN, CDE [2]; Robert I. Berkowitz, MD [3]; Seth Braunstein, MD, PhD [3] ; Gary Foster, PhD [3]; Henry Glick, PhD [3]; Shiriki Kumanyika, PhD, RD, MPH [3]; Stanley S. Schwartz, MD [3] ; Yuliis Bell, BA; Raymond Carvajal, PsyD; Helen Chomentowski; Renee Davenport; Anthony Fabricatore, PhD; Lucy Faulconbridge, PhD; Louise Hesson, MSN, CRNP; Nayyar Iqbal, MD; Robert Kuehnel, PhD; Patricia Lipschutz, MSN; Monica Mullen, RD, MPH

University of Pittsburgh John M. Jakicic, PhD[1]; David E. Kelley, MD[1]; Jacqueline Wesche-Thobaben, RN, BSN, CDE[2]; Lewis H. Kuller, MD, DrPH[3]; Andrea Kriska, PhD[3]; Amy D. Rickman, PhD, RD, LDN[3]; Lin Ewing, PhD, RN[3]; Mary Korytkowski, MD[3]; Daniel Edmundowicz, MD[3]; Rose Salata, MD[3]; Rebecca Danchenko, BS; Tammy DeBruce; Barbara Elnyczky; David O. Garcia, MS; Patricia H. Harper, MS, RD, LDN; Susan Harrier, BS; Dianne Heidingsfelder, MS, RD, CDE, LDN; Diane Ives, MPH; Juliet Mancino, MS, RD, CDE, LDN; Lisa Martich, MS, RD; Tracey Y. Murray, BS; Karen Quirin; Joan R. Ritchea; Susan Copelli, BS, CTR

The Miriam Hospital/Brown Medical School Rena R. Wing, PhD[1]; Renee Bright, MS[2]; Vincent Pera, MD[3]; John Jakicic, PhD[3]; Deborah Tate, PhD[3]; Amy Gorin, PhD[3]; Kara Gallagher, PhD[3]; Amy Bach, PhD; Barbara Bancroft, RN, MS; Anna Bertorelli, MBA, RD; Richard Carey, BS; Tatum Charron, BS; Heather Chenot, MS; Kimberley Chula-Maguire, MS; Pamela Coward, MS, RD; Lisa Cronkite, BS; Julie Currin, MD; Maureen Daly, RN; Caitlin Egan, MS; Erica Ferguson, BS, RD; Linda Foss, MPH; Jennifer Gauvin, BS; Don Kieffer, PhD; Lauren Lessard, BS; Deborah Maier, MS; JP Massaro, BS; Tammy Monk, MS; Rob Nicholson, PhD; Erin Patterson, BS; Suzanne Phelan, PhD; Hollie Raynor, PhD, RD; Douglas Raynor, PhD; Natalie Robinson, MS, RD; Deborah Robles; Jane Tavares, BS

The University of Texas Health Science Center at San Antonio Steven M. Haffner, MD[1]; Helen P. Hazuda, PhD[1]; Maria G. Montez, RN, MSHP, CDE[2]; Carlos Lorenzo, MD[3]; Charles F. Coleman, MS, RD; Domingo Granado, RN; Kathy Hathaway, MS, RD; Juan Carlos Isaac, RC, BSN; Nora Ramirez, RN, BSN

VA Puget Sound Health Care System / University of Washington Steven E. Kahn, MB, ChB[1]; Anne Murillo, BS[2]; Robert Knopp, MD[3]; Edward Lipkin, MD, PhD[3]; Dace Trence, MD[3]; Elaine Tsai, MD[3]; Basma Fattaleh, BA; Diane Greenberg, PhD; Brenda Montgomery, RN, MS, CDE; Ivy Morgan-Taggart; Betty Ann Richmond, MEd; Jolanta Socha, BS; April Thomas, MPH, RD; Alan Wesley, BA; Diane Wheeler, RD, CDE

Southwestern American Indian Center, Phoenix, Arizona and Shiprock, New Mexico William C. Knowler, MD, DrPH[1]; Paula Bolin, RN, MC[2]; Tina Killean, BS[2]; Cathy Manus, LPN[3]; Jonathan Krakoff, MD[3]; Jeffrey M. Curtis, MD, MPH[3]; Sara Michaels, MD[3]; Paul Bloomquist, MD[3]; Peter H. Bennett, MB, FRCP[3]; Bernadita Fallis RN, RHIT, CCS; Diane F. Hollowbreast; Ruby Johnson; Maria Meacham, BSN, RN, CDE; Christina Morris, BA; Julie Nelson, RD; Carol Percy, RN; Patricia Poorthunder; Sandra Sangster; Leigh A. Shovestull, RD, CDE; Miranda Smart; Janelia Smiley; Teddy Thomas, BS; Katie Toledo, MS, LPC

University of Southern California Anne Peters, MD[1]; Siran Ghazarian, MD[2]; Elizabeth Beale, MD[3]; Kati Konersman, RD, CDE; Brenda Quintero-Varela; Edgar Ramirez; Gabriela Rios, RD; Gabriela Rodriguez, MA; Valerie Ruelas MSW, LCSW; Sara Serafin-Dokhan; Martha Walker, RD

Coordinating Center

Wake Forest University Mark A. Espeland, PhD[1]; Judy L. Bahnson, BA, CCRP[3]; Lynne E. Wagenknecht, DrPH[3]; David Reboussin, PhD[3]; W. Jack Rejeski, PhD[3]; Alain G. Bertoni, MD, MPH[3]; Wei Lang, PhD[3]; Michael S. Lawlor, PhD[3]; David Lefkowitz, MD[3]; Gary D. Miller, PhD[3]; Patrick S. Reynolds, MD[3]; Paul M.

Ribisl, PhD[3]; Mara Vitolins, DrPH[3]; Daniel Beavers, PhD[3]; Haiying Chen, PhD, MM[3]; Dalane Kitzman, MD[3]; Delia S. West, PhD[3]; Lawrence M. Friedman, MD[3]; Ron Prineas, MD[3]; Tandaw Samdarshi, MD[3];Kathy M. Dotson, BA[2]; Amelia Hodges, BS, CCRP[2]; Dominique Limprevil-Divers, MA, MEd[2]; Karen Wall[2]; Carrie C. Williams, MA, CCRP[2]; Andrea Anderson, MS; Jerry M. Barnes, MA; Mary Barr; Tara D. Beckner; Cralen Davis, MS; Thania Del Valle-Fagan, MD; Tamika Earl, Melanie Franks, BBA; Candace Goode; Jason Griffin, BS; Lea Harvin, BS; Mary A. Hontz, BA; Sarah A. Gaussoin, MS; Don G. Hire, BS; Patricia Hogan, MS; Mark King, BS; Kathy Lane, BS; Rebecca H. Neiberg, MS; Julia T. Rushing, MS; Valery S. Effoe, MD,MS; Michael P. Walkup, MS; Terri Windham

Central Resources Centers

DXA Reading Center, University of California at San Francisco Michael Nevitt, PhD[1]; Ann Schwartz, PhD[2]; John Shepherd, PhD[3]; Michaela Rahorst; Lisa Palermo, MS, MA; Susan Ewing, MS; Cynthia Hayashi; Jason Maeda, MPH

Central Laboratory, Northwest Lipid Metabolism and Diabetes Research Laboratories Santica M. Marcovina, PhD, ScD[1]; Jessica Hurting[2];
John J. Albers, PhD[3], Vinod Gaur, PhD[4]

ECG Reading Center, EPICARE, Wake Forest University School of Medicine
Elsayed Z. Soliman MD, MSc, MS[1]; Charles Campbell [2]; Zhu-Ming Zhang, MD[3]; Mary Barr; Susan Hensley; Julie Hu; Lisa Keasler; Yabing Li, MD

Diet Assessment Center, University of South Carolina, Arnold School of Public Health, Center for Research in Nutrition and Health Disparities
Elizabeth J Mayer-Davis, PhD[1]; Robert Moran, PhD[1]

Hall-Foushee Communications, Inc.
Richard Foushee, PhD; Nancy J. Hall, MA

Federal Sponsors

National Institute of Diabetes and Digestive and Kidney Diseases Mary Evans, PhD; Barbara Harrison, MS; Van S. Hubbard, MD, PhD; Susan Z. Yanovski, MD

National Heart, Lung, and Blood Institute Lawton S. Cooper, MD, MPH; Peter Kaufman, PhD, FABMR; Mario Stylianou, PhD

Centers for Disease Control and Prevention Edward W. Gregg, PhD; Ping Zhang, PhD

---

[1] Principal Investigator
[2] Program Coordinator
[3] Co-Investigator
All other Look AHEAD staffs are listed alphabetically by site.
